# Effect of different synbiotic administration methods on growth, carcass characteristics, ileum histomorphometry, and blood biochemistry of Cobb-500 broilers

**DOI:** 10.14202/vetworld.2024.1238-1250

**Published:** 2024-06-08

**Authors:** Arjun Acharya, Bhuminand Devkota, Hom Bahadur Basnet, Shanker Raj Barsila

**Affiliations:** 1 Department of Animal Nutrition and Fodder Production, Agriculture and Forestry University, Faculty of Animal Science, Veterinary Science and Fisheries, Rampur, Chitwan, 00977 Nepal; 2 Department of Theriogenology, Agriculture and Forestry University, Faculty of Animal Science, Veterinary Science and Fisheries, Rampur Chitwan, 00977 Nepal; 3 Department of Veterinary Microbiology, Agriculture and Forestry University, Faculty of Animal Science, Veterinary Science and Fisheries, Rampur Chitwan, 00977 Nepal; 4 Department of Animal Nutrition and Fodder Production, Faculty of Animal Science, Veterinary Science and Fisheries, Rampur, Chitwan, Nepal

**Keywords:** growth performance, gut health, histomorphometry, *in ovo*, synbiotic

## Abstract

**Background and Aim:**

To combat enteric infections and antibiotic resistance in the poultry industry, researchers seek alternatives such as probiotics, prebiotics, and synbiotics as growth promoters. Synbiotics support probiotic growth through the supply of essential nutrients. The study’s objectives were to assess the most effective delivery methods for synbiotics and evaluate their growth, histomorphometric, and hematological impacts on Cobb-500 broilers.

**Materials and Methods:**

Two studies, independently conducted, employed a completely randomized design. One hundred and eighty viable eggs in the first trial were assigned to three groups: Control (T1), sterile water (T2), and synbiotic in sterile water (T3). On the 21^st^ day of hatching, hatchability, day-old body weights, and ileum samples for histomorphometric analysis were recorded. In the second trial, out of 500 viable eggs, 200 eggs were fed *in ovo* with synbiotics (PoultryStar® sol, Biomin Singapore Pte Ltd, Singapore) on 17.5 days and 300 were set aside without *in ovo* injection. The treatments were control (T1), in water synbiotic (T2), *in ovo* synbiotic (T3), combination of *in ovo* synbiotic and synbiotic in feed (T4), and synbiotic in feed only (T5). On 21 and 42 days, blood, ileum, and visceral organ samples were collected for laboratory analysis. Data on weight gain, daily feed intake, and water consumption were recorded for 42 days.

**Results:**

The initial experiment’s results revealed a decrease in hatchability, slight weight increase, and significant intestinal morphological changes with the use of an *in ovo* synbiotic. Applying synbiotic through various methods in the second trial yielded better growth results, lower blood cholesterol, and significantly longer (p < 0.05) villi on 21 days.

**Conclusion:**

Using the *in ovo* method to administer synbiotics lowered hatchability. Use of synbiotics with any method or in combination enhances growth, ileum structure, dressing yield, feed efficiency, and cholesterol levels in blood. Synbiotics enhance gut health and overall performance in broilers when used through diverse approaches.

## Introduction

Among all agricultural commodities, poultry holds the fastest growing rate of production [1]. With the rise in world population, there is an increasing demand for poultry products because they are cost-effective and widely recognized as a popular protein source [2]. The challenge for the poultry industry is to eliminate or control foodborne and zoonotic pathogens. The concern over public health risks related to consuming foods with high antibiotic residues is an emergent matter [3]. In poultry farming, antibiotics and other traditional antimicrobials are commonly used to prevent and treat diseases. Misuse of these substances encourages antimicrobial drug resistance, posing major public health concerns [4]. With the rise of concern over antibiotic resistance and the ban of antibiotic growth promoters in numerous countries [5, 6], the quest for alternatives in poultry production has intensified. In poultry production, several options have been considered, such as prebiotics, probiotics, synbiotics, organic acids, essential oils, enzymes, and emerging novel compounds. Probiotics, prebiotics, and their combination (synbiotics) have become increasingly significant substitutes [7]. Enteric infections significantly impact poultry health by lowering productivity, increasing mortality, and potentially contaminating human meat consumption [8].

Birds, in their quest for nutrients, unwittingly consume harmful bacteria from their host, potentially infecting their small intestines [9]. Prebiotics and probiotics, which modify gut microbiota and the immune system, can prevent pathogen colonization and, thereby, enteric illnesses in poultry farming [10]. Probiotics need a prebiotic to survive in the digestive tract and become resilient to environmental challenges. Synbiotics are essential to support probiotic growth because they provide the substrate required for fermentation [11] and create a synergistic effect [12]. The addition of synbiotic to broiler diets enhanced body weight, growth, feed efficiency, and carcass yield by improving the condition of the intestinal tract, thereby increasing villi height (VD), crypt depth (CD), and the overall absorptive area. 0.1% and 0.15% synbiotic supplementation to broilers’ diets from day 1 to day 42 enhanced their body weight gain and feed conversion ratio (FCR) [13]. Adding synbiotics to feed has been proven to lower coccidian oocyst numbers, enhancing broiler gut health. The administration of synbiotics resulted in shallower CDs in the ileum and duodenum and taller villi in the duodenum, jejunum, and ileum for broilers [14]. Broilers’ body weight and hemato-biochemical profile have been improved by probiotics and synbiotics [15]. A synbiotic, rather than a probiotic, is more effective in enhancing growth and health in broilers [16]. The synbiotic-supplemented diet enhanced various parameters, such as daily weight gain, feed efficiency, villus height, and villus: crypt ratio on 21 and 42 days, surpassing the control group [17].

Effective protection against environmental and disease challenges in chickens relies on early bacterial colonization of the gut. The gut microbiota, pathogens, and the host’s intestinal system form a crucial symbiotic relationship necessary for gastrointestinal maturation and rapid growth in broiler chickens. Hatching chick losses and economic damage can result from microbial contamination during pre-hatch development [18]. To ensure overall health, prevent pathogen colonization, and promote immune development, synbiotic is delivered *in ovo* to the embryo, resulting in early colonization by beneficial microbes.

The *in ovo* method is employed during the early embryonic stages due to its ability to prevent diseases in later life and boost productivity through environmental exposure and nutritional manipulation [19]. Inclusion of bioactive compounds in poultry feed poses several challenges. The manufacturing process’ high temperatures could degrade the product if it is included in the feed. The compounds’ nutritional value and bioactive properties could be altered. The effects of watering devices and water quality on the biological efficacy of substances in drinking water are significant [20, 21].

The effectiveness of different synbiotic administration methods remains uncertain despite numerous discoveries detailing their health benefits. These studies [22–26] demonstrated enhanced poultry performance and health from synbiotic administration. Synbiotic’s application methods vary, influenced by the intended product outcomes [27, 28]. In-feed/in-water supplementation provides ongoing exposure when used, but *in-ovo* injection allows for early-life programming of the gut microbiome and immune system [28, 29]. The effectiveness of synbiotic administration methods on poultry growth remains undefined.

To reach optimal outcomes in the poultry industry, evaluation of various methods and employing proficient management are essential. New strategies are being proposed to optimize the efficacy of multiple alternatives while decreasing the use of antibiotics in food animal production [2]. This research aims to explore the impact on growth and intestinal histomorphology of various synbiotic feeding methods and their combinations.

## Materials and Methods

### Ethical approval

The study was approved by the Agriculture and Forestry University Institutional Review Board, Nepal (Approval number AFU-FWA00031653).

### Study period and location

The study was conducted from November to December 2022 in the experimental shed of Fewa Group of Poultry Industries Pvt. Ltd. Pokhara, Nepal. The hatching eggs and day-old chicks were collected from the same hatchery for *in ovo* operation and other experimental procedures.

### Preparation of the synbiotic solution

Dry synbiotic powder (PoultryStar®sol, Biomin Singapore Pte Ltd, Singapore) was used as feed material. PoultryStar® me containing prebiotic fructooligosaccharides and probiotic bacteria (Enterococcus sp., Pediococcus sp., Bifidobacterium sp., and Lactobacillus sp.) containing a minimum of 2 × 10^11^ colony-forming unit/kg (2 x 10 power 11), Fructooligosaccharides and the microorganisms *Enterococcus faecium*, *Pediococcus acidilactici*, *Bifidobacterium animalis*, and *Lactobacillus reuteri* comprise the prebiotics and probiotic source in PoultryStar® sol containing a minimum of 5.0 × 10^12^ colony-forming unit/kg. 20 g of synbiotic was diluted in 500 mL of sterile water for every 1000 chicks, according to the manufacturer’s instructions. In this experiment, 5 g of synbiotic was dissolved in 125 mL of sterile warm water, heated to 37.5°C, for use in the *in ovo* procedure.

### *In ovo* method for feeding

300 fertilized eggs were collected from 35-week-old Cobb-500 broiler breeders with comparable weight (>70% hatchability) for the *in ovo* method study. 75% ethanol was used to clean the eggshells before incubation, as per the protocol. 180 viable eggs, selected on 17 days, were distributed into three treatment groups for the *in ovo* process. The control group remains untreated, while the other groups were treated with synbiotic and sterile water. Each treatment underwent weighing, numbering, and standard temperature incubation. Approximately 420 h of incubation, the *in ovo* operation was carried out under sterile conditions and maintained cleanliness. 1 mm holes were made in each egg and 0.5 ml of the *in ovo* supplement was injected into the amniotic cavities. The *in ovo* process, lasting 30 min, was followed by the eggs’ return to the incubator. The control group’s eggs were similarly incubated. Then, the eggs were transferred to the hatcher for further development. On 21^st^ day- day old chicks from various groups were examined, weighed and ileum samples were taken during postmortem examination and egg shells were broken in unhatched eggs to determine the causes of mortality. The specifics of the first experiment’s treatment are presented in Table-1.

**Table-1:** Description of *in ovo* treatments applied to Cobb-500 broiler breeders’ fertile eggs on 420 h in experiment no. 1

Treatment	Description
T1 = Control group	Without supplements
T2 = *In-ovo* sterile water	Use of pre-warmed sterile water at 37.5°C
T3 = Synbiotics	Synbiotics dissolved in prewarmed sterile water 37.5°C

### Experimental design, diet, and management

The second study assessed the synbiotic’s impact on blood chemistry, intestinal structure, carcass features, and growth efficiency. Chicks received synbiotic through a combination of *in ovo* injection, feed, and water. 500 fertile eggs were obtained, with 200 of these being fed *in ovo* on 17.5 days and the remaining 300 incubated without feeding. Twenty-one days after the hatching, 120 *in ovo* chicks and 180 normal chicks, altogether 300 eggs were chosen for the study. 30-floor pens, housing five treatments with six replicates each, were randomly assigned to the hatchlings and equipped with 10-chick compartments. Chick placement was done using a completely randomized design. The chicks were raised under optimal commercial broiler conditions, ensuring the correct levels of light, humidity, and temperature. The corn and soybean meal mash feed produced alongside starter, grower, and finisher feed on the same farm is presented in Table-2. The feeding formulation’s nutritional content met Cobb-500 broiler standard guidelines. At Global Lab in Chitwan, Nepal, nutrient analyses were performed on samples from each age formulation. The calculated and laboratory analysis of feed samples used in the experiment are presented in Tables-3 and 4, respectively. The recommended dosages of Synbiotic PoultryStar® sol and Synbiotic PoultryStar® me (Biomin®) were administered in chickens’ drinking water and feed, respectively, through the manufacturer’s instructions (20 g/1000 chicks for the sol through water, 0.5 kg per ton of feed for the me through feed). Birds were raised in deep litter for 42 days, with unlimited food and water access. Table-5 displays the experimental groups involved in the research.

**Table-2:** Diet composition used for the feeding trial in experiment no. 2

Ingredients	Quantity of the ingredients (in kg)			Ingredients	Quantity of the ingredients (in kg)		
	
	B0	B1	B2		B0	B1	B2
Maize	550	600	633.5	L-Lysine HCL	3.2	3.2	3
Rice Polish	40.5	70	60	L-Threionine	0.8	1.4	1.5
Oil-soy	24	30	32	Choline Chloride	1	1	1
Soya DOC Hi-pro	345.8	262	238	Lipidin	0.5	0.5	0.5
Salt	2.0	2.0	1.5	Toxin Binder	1	1	1
Sodium Bicarbonate	2.5	2.3	2.7	Trace mineral Mixture*	1.3	1.3	1.3
Di-Calcium Phosphate	11	10	8	Liver tonic	0.5	0.5	0.5
Phytase (5000FTU)	0.1	0.1	0.1	Vitamin Premix**	0.5	0.5	0.5
Limestone Powder	12	11	12	Acidifier	1	1	1
DL-Methionine	2.2	2.1	1.8	Antioxidant	0.1	0.1	0.1

* Tracemin CB (Venky’s India Private Limited, Pune). Each 1 kg TraceMin-CB contains Manganese=100 g, Zinc=80 g, Iron=90.0 g, Copper=15.0 g, copper=15.0 g, iodine=2.0 g, selenium=300 mg.

** Vitamin Premix-Each 500 g contains Vitamin A 13.50 MIU, Vitamin D3 04.50MIU, Vitamin E 60.0 g, Vitamin K 03.50g, Vitamin B1 03.50 g, Vitamin B2 08.00 g, Vitamin B6 03.50 g, Vitamin B12 0.02 g, Niacin 60.00 g, Calcium pantothenate 14.50 g, Folic acid 02.25 g, Biotin 0.145 g, Vitamin C 90.00 g, Organic Nutritive Carrier QS. B0-Broiler starter diet for 0–14 days, B1-Broiler grower diet for 15–28 days, B2-Broiler finisher diet 29–42 days

**Table-3:** Calculated analysis of nutrients (%) in the diet

Nutrient name	Feed items			Nutrient Name	Feed items		
	
	B0	B1	B2		B0	B1	B2
M.E (kcal/kg)	3026	3101	3150	Chloride, %	0.18	0.18	0.15
Crude Protein, %	22.19	19.03	18.07	Potassium, %	0.91	0.80	0.75
Dig. Lysine, %	1.28	1.09	1.02	Dig. Arginine, %	1.34	1.11	1.04
Dig. Methionine, %	0.49	0.45	0.41	Dig. Tryptophan, %	0.21	0.17	0.16
Dig. Meth+Cystine, %	0.86	0.80	0.76	Dig. Threionine, %	0.74	0.68	0.67
Calcium, %	0.93	0.85	0.83	Dig. Isoleucine, %	0.79	0.66	0.62
Availbale Phosphorus, %	0.45	0.42	0.38	Dig. Valine, %	0.86	0.73	0.70
Sodium, %	0.16	0.16	0.15	Linoleic Acid, %	2.44	2.84	2.95
Crude Fiber, %	4.06	4.25	4.20				

B0 for 0–14 days, B1 for 15–28 days, and B2 for >29 days

**Table-4:** Proximate composition of the feed samples used to feed broilers during the experiment no. 2.

Parameters	Starter (B0)	Grower (B1)	Finisher (B2)
Crude protein, %	22.09	19.13	18.34
Ash, %	5.67	5.16	5.44
Fat, %	5.16	5.70	6.27
Crude fiber, %	3.42	3.46	3.24
Moisture, %	11.93	11.53	11.33

Test performed at Global Lab, Chitwan by NIR, serial no. 101920. B0-Broiler starter diet for 0–14 days, B1-Broiler grower diet for 15–28 days, B2-Broiler finisher diet 29–42 days

**Table-5:** Treatment details used in experiment 2.

Treatments	Description
T1 = Control	No additives
T2 = Synbiotics in water	PoultryStar®sol through water (20 g/1000chicks)
T3 = *In ovo* synbiotics	PoultryStar®sol at 20 g/1000 chicks through *in ovo*
T4 = *In ovo* synbiotics + synbiotics in feed	PoultryStar®sol through *in ovo* and PoultryStar®me through feed regularly at 0.5kg per ton of feed
T5 = Synbiotics in feed	PoultryStar® me through feed at 0.5 kg per ton of feed

PoultryStar®sol-. Prebiotics fructooligosaccharides and the probiotic strains *Enterococcus faecium*, *Pediococcus acidilactici*, *Bifidobacterium animalis*, and *Lactobacillus reuteri*. The product contains a minimum of 5.0 × 10^12^ Colony-forming unit/kg, manufactured by Biomin Singapore Pte Ltd, Singapore)

### Experimental procedure and sample collection

The experimental chicks were housed in an open system. During sampling, every step was taken to ensure minimal stress and pain for the birds. The investigator knew only the details of the treatment. Samples were decoded and analyzed after the laboratory reports were obtained and histomorphometry and blood biochemistry tests were completed. Chicks were weighed daily for 42 consecutive days at the same time. Daily records were taken for body weight, feed intake, and water consumption. Weekly weight gain, feed consumption, and FCR were calculated. Daily feed waste and bird’s mortality were accounted for in the recorded feed consumption. Samples for the histomorphometric study were collected on days 21 and 42 for all treatments. 18 birds per treatment underwent organ weighing and autopsy on days 21 and 42. 10% formalin was used to preserve the ileum samples before they were transported to the laboratory. On day 42, 18 birds from each treatment group had their blood sample (3 ml from each bird) drawn for serum biochemistry and hematological analysis. The levels of total protein, albumin, glucose, and total cholesterol in serum were determined.

### Histomorphometric study

At Star Diagnostic Laboratory, Pokhara, Nepal, histological slides were prepared for study from 18 chicks of each treatment group. Chicks and birds were decapitated. 1 cm intestinal samples (posterior to the Meckel’s diverticulum and anterior to the ileocecal junction) were collected, cleaned, fixed in 10% formalin, and kept at the laboratory for 48 h. The samples were dehydrated using successively increasing concentrations of alcohol (70%, 80%, 90%, 95%, and 100%) at 1 h intervals, then cleared in xylene for 2 h each. The tissues were then processed by infiltrating with molten wax, embedding in molten paraffin, and labeled. 5 μ thick sections were cut using a microtome knife.

The tissue sections were mounted on clean glass slides, air-dried, and stained with hematoxylin and eosin. The slides were observed under a 400× magnification light microscope (Coslab Coaxial Research Binocular Microscope, Coslab, India, Model-std 9). Microscopic images were captured using a Samsung Galaxy M62 camera (model SM-M6257FDS, Samsung India Electronics Pvt. Ltd., India). The camera was adjusted to a coarse adjustment and fixed to the eyepiece with clamps. Photographs were taken at various tissue sample sites with fine adjustments.

A slide from Erma Inc. (code: Esm11/1000/0509/859, Tokyo, Japan), featuring a 0.01 mm linear scale with 100 divisions, and each division being 10 μm, was employed as a reference for measurement. The image of the micrometer scale on the stage of the microscope used for capturing tissue samples was employed to calculate VD and CD by setting it as the reference scale in ImageJ software (https://imagej.en.softonic.com/). In the intestinal cross-sections of each sample, at least, three intact and well-aligned villus crypt units were observed, and their corresponding averages were recorded.

### Blood biochemistry

On day 42, 90 avian blood samples were collected (3 birds per pen, 30 pens, 6 replications, 5 treatments, 3 mL each) for hematological analysis and serum extraction for serum biochemistry. 1 h at room temperature (20–25°C), blood samples were left to clot in non-ethylenediaminetetraacetic acid tubes. The serum was separated from the remainder by centrifugation at 1006× *g* for 20 mins. The serum sample was stored at −20°C before chemical analysis. The samples were analyzed for total cholesterol, albumin, glucose, and protein after being thawed. The Star Diagnostic Laboratory in Pokhara, Nepal, carried out all the hematological tests.

### Visceral organ examination

Six birds were randomly selected on days 21 and 42, each from six replications of the treatment for determination of organ weights, following their decapitation. 5 min after the bleeding, the birds underwent scalding, defeathering, and evisceration, involving removal of their heads, necks, and legs. The weights of the liver (excluding gall bladder), proventriculus, heart, spleen, Fabricius bursa, small intestine, two caeca, and visible fat (surrounding viscera, gizzard, and subcutaneous fat) were all measured. The organ weights were expressed as a percentage of the live bird weight. The weights and relative proportions of the breast muscle, drumsticks, gizzard, and proventriculus were recorded and calculated.

### Mortality

Mortality, including the cause and adjustments to feed intake and water consumption were recorded during the experimental period.

### Statistical analysis

One-way analysis of variance in R Statistics (version 4.3.2) synchronized with R Studio (https://rstudio.com/products/rstudio/download/#download) was used for statistical analysis. To assess variations between treatments, Duncan’s multiple range test was used when the p-value was 0.05. The arithmetic means and standard error of the mean are presented.

## Results

### Effect of *in ovo* feeding on hatchability, body weight, and histomorphometry

The findings from *in ovo* feeding are presented in Table-6. *In ovo* treatments did not impact hatchability. The distilled water and synbiotic treatment resulted in weight difference (p < 0.05) compared to the non *in ovo* group in the experiment. In the synbiotic treatment group, villi length (288.60 μm) and CD (49.50 μm) were significantly longer (p < 0.05) than the other treatment groups. The synbiotic treatment had no effect on the villus to CD ratio.

**Table-6:** Effect of *in ovo* synbiotic feeding on hatchability and ileal histomorphometry of broilers.

Treatments	Initial egg weight (g)	17.5 days weight (g)	Day old weight (g)	Hatchability (%)	VH (μm)	CD (μm)	VH/CD Ratio
T1	62.38	55.52	42.00^b^	93.33	260.14^b^	46.05^a^	5.66
T2	62.50	55.51	42.41^a^	88.33	269.11^b^	47.26^b^	5.70
T3	62.46	55.49	42.55^a^	89.48	288.60^a^	49.50^b^	5.84
Average	62.44	55.51	42.32	90.38	272.61	47.61	5.73
CV, %	0.20	0.18	0.42	8.32	3.63	3.43	5.88
p-value	0.264	0.845	< 0.001	0.500	< 0.001	< 0.001	0.650
SEM (±)	0.04	0.01	0.16	1.51	8.40	0.70	0.44

CV=Coefficient of variation, SEM=Standard error of the mean, T1=Normal chicks, T2=Distilled water feed *in ovo*, synbiotics T3=Synbiotics *in ovo*. In the same row, values with the same letter superscripts indicate no significant difference (p > 0.05), while values with different superscripts in the column indicate significant difference (p < 0.05), VH=Villi height, CD=Crypt depth.

### Mortality and feed consumption

Synbiotics improve overall health in organisms, indicated by decreased mortality and increased production. The data on mortality and feed consumption are presented in Tables-7 and 8. During the experiment, neither mortality nor feed consumption was influenced by any treatment.

**Table-7:** Mortality pattern of broilers during the feeding experiment.

Treatments	Day 7	Day 14	Day 21	Day 28	Day 35	Day 42
T1	0.00	0.33	0.00	0.00	0.00	0.00
T2	0.16	0.50	0.33	0.00	0.00	0.16
T3	0.66	0.50	0.33	0.00	0.33	0.00
T4	0.66	0.66	0.00	0.00	0.33	0.00
T5	0.00	0.33	0.16	0.16	0.33	0.00
Average	0.30	0.46	0.16	0.03	0.20	0.03
CV, %	205.4	154.52	281.42	547.72	200	547.71
p-value	0.152	0.922	0.563	0.426	0.316	0.426
SEM(±)	0.15	0.06	0.03	0.08	0.03	0.07

SEM=Standard error of the mean, T1=Control, T2=Synbiotics through water, T3=*In ovo* synbiotics, T4=In-ovo synbiotics+synbiotics through feed, T5=Synbiotics through feed

**Table-8:** Weekly feeding pattern of broilers administered synbiotics by different methods.

Treatments	Week 1	Week 2	Week 3	Week 4	Week 5	Week 6
T1	145.58	325.52	552.15	753.00	966.92	1225.72
T2	148.25	333.12	598.35	805.19	963.72	1190.10
T3	151.30	347.81	584.82	774.89	980.40	1211.11
T4	147.37	333.60	577.36	774.44	949.38	1202.03
T5	149.82	330.38	560.26	753.14	969.49	1245.16
Average	148.46	334.09	574.58	772.13	965.98	1214.82
CV, %	2.92	5.28	5.99	11.28	7.05	4.28
p-value	0.217	0.286	0.171	0.833	0.956	0.419
SEM (±)	0.99	3.72	8.32	9.57	5.01	9.56

CV=Coefficient of variation, SEM=Standard error of the mean, T1=Control, T2=Synbiotics through water, T3=*In ovo* synbiotics, T4=*In ovo* synbiotics + synbiotics through feed, T5=Synbiotics through feed

### Weight gain and FCR

**Figure-1:**
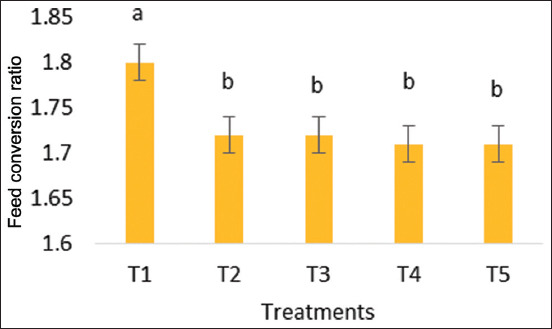
Feed conversion ratio at 6 weeks of age (T1=No use of supplements, T2=synbiotic through water, T3=*In ovo* synbiotic, T4=*In ovo* synbiotic and synbiotic through feed, and T5=Synbiotic through feed only).

**Figure-2:**
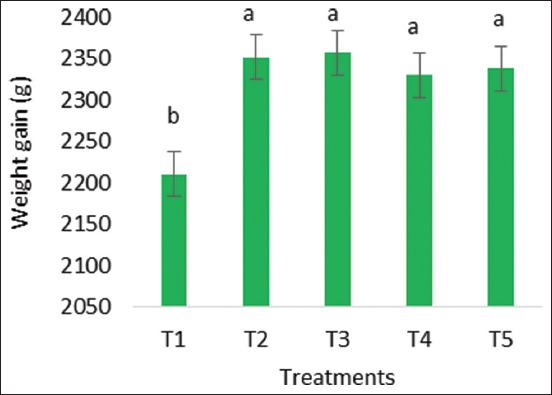
Weight of broilers at 6 weeks of age (T1=No use of supplements, T2=synbiotic through water, T3=*In ovo* synbiotic, T4=*In ovo* synbiotic and synbiotic through feed, and T5=Synbiotic through feed only).

At 6 weeks, both weight gain and FCR were significantly different from each other (p < 0.05), as shown in Figures-1 and 2. In the 6^th^ week, the broiler weight gain was significantly greater in the synbiotic-treated group compared to the control group. On 42 days, the treated group gained significantly more weight (above 2300 g) than the control group (2209.83 g), while the FCR in the control group was higher (1.8) than in the treated group (around 1.7). In all synbiotic treatment groups, the FCR was significantly lower (p < 0.05) than the control group at the 6^th^ week of age.

### Blood parameters

The levels of blood glucose, serum albumin, total protein, and total cholesterol are presented in Table-9. In the synbiotic-treated group, the mean total cholesterol level was 119.33 mg/dL, lower than in the control group (p < 0.05). The blood sugar, serum albumin, and total protein levels were comparable across the groups.

**Table-9:** Blood biochemistry of broilers administered synbiotics by different methods.

Treatments	Blood glucose (mg/dL)	Serum albumin (g/dL)	Total protein (g/dL)	Total cholesterol (mg/dL)
T1	237.33	1.06	3.16	119.33^a^
T2	233.00	1.07	3.10	111.17^bc^
T3	224.50	1.15	3.25	113.00^b^
T4	238.17	1.12	3.27	106.50^c^
T5	240.67	1.13	3.40	113.83^b^
Average	234.73	1.10	3.24	106.83
CV, %	5.27	9.57	8.49	3.70
p-value	0.210	0.526	0.400	0.001
SEM(±)	2.84	0.02	0.05	2.08

CV=Coefficient of variation, SEM=Standard error of the mean, control, T1=Control, T2=Synbiotics through water, T3=*In ovo* synbiotics, T4=*In ovo* synbiotics in feed, T5=Synbiotics through the feed. In the same row, values with no letter or the same letter superscript indicate no significant difference (p > 0.05), while values with different superscripts (a, b, and c) indicate a significant difference (p < 0.05)

### Relative weight of visceral organs

Percentages of visceral organ weights on days 21 and 42 are presented in Table-10. 21-day-old experimental groups had an average dressing percentage of 62.61%. The dressing percentage rose to an average of 74.39% after 42 days. In the control group, dressing percentage was lower (72.72%) than in the treatment group where synbiotic was administered via water (75.18%). On 21 days of age, the liver, gizzard, and intestine of treatment groups weighed significantly more (p < 0.05) compared to the control group. In the control group, the spleen’s relative weight (0.12%) was smaller than in the synbiotic supplemented group. On 42 days, the liver and intestine weighed less relatively in both the control and treatment groups. In the synbiotic used group, the intestine’s relative weight was significantly lower (3.71%) compared to the control, with the lowest being 3.1% in the group administered synbiotic *in ovo* (p < 0.05). The spleen of the synbiotic-treated *in ovo* group was smaller than that of the control group. The synbiotic treatment resulted in a reduced relative weight of the bursa in birds, implying its role in immunological functions. In synbiotic-treated poultry, the proportion of less edible parts to overall weight was smaller than in the control group, suggesting the positive impact of synbiotic on poultry feeding.

**Table-10:** Relative organ weight as a percentage of live weight of birds on 21 and 42 days after administration of synbiotics using different methods.

Treatments	T1		T2		T3		T4		T5		Average		CV, %		p-value		SEM	
								
Days	42	21	42	21	42	21	42	21	42	21	42	21	42	21	42	21	42	21
Live Weight, g	2209.83^b^	807.79	2351.1^a^	811.94	2356.23^a^	808.52	2329.7^a^	781.67	2337.27^a^	819.42	2316.83	805.87	3.27	5.25	0.014	00.609	27.16	6.39
Dressing	72.72^b^	61.99	75.18^a^	62.46	74.76^a^	62.97	75.16^a^	62.62	74.12^ab^	63	74.39	62.61	1.74	1.88	00.015	0.573	0.460	0.19
Liver	2.19^a^	3.04^b^	1.97^b^	3.8^a^	1.92^b^	3.69^a^	2.04^ab^	3.12^b^	2.03^ab^	3.00^b^	2.03	3.33	6.41	11.3	0.015	0.001	0.046	0.17
Gizzard	2.28^a^	3.22^b^	2.12^ab^	3.82^a^	1.87^b^	3.57^ab^	2.05^ab^	3.58^ab^	2.28^a^	3.20^b^	2.12	3.47	10.14	8.95	0.015	00.001	0.076	0.12
Heart	0.62^a^	0.61	0.55^ab^	0.76	0.47^c^	0.71	0.5^bc^	0.66	0.58^a^	0.55	0.54	0.66	11.53	22.19	0.001	0.133	0.028	0.04
Spleen	0.12^a^	0.12^c^	0.12^a^	0.15^a^	0.09^b^	0.14^ab^	0.09^b^	0.12^bc^	0.13^a^	0.11^c^	0.11	0.13	18.97	13.19	00.006	0.001	0.008	0.01
Bursa	0.09^a^	0.15	0.08^ab^	0.14	0.07^bc^	0.13	0.07^c^	0.17	0.09^a^	0.11	0.83	0.14	11.66	39.99	0.019	0.529	0.003	0.01
Intestine	3.71^a^	5.37^c^	3.35^b^	5.87^bc^	3.29^b^	6.20^ab^	3.10^b^	6.71^a^	3.36^b^	6.28^ab^	3.36	6.09	6.36	9.83	<0.001	0.009	0.102	0.22
Ceca	0.05^a^	0.57	0.48^ab^	0.55	0.41^b^	0.59	0.41^b^	0.6	0.44^b^	0.65	0.45	0.59	13.26	21.48	0.005	0.707	0.024	0.02
Visible Fat	3.00^a^	1.77	2.54^ab^	2.17	2.33^b^	2.58	2.33^b^	2.12	2.62^ab^	1.68	2.56	2.06	18.27	28.81	0.012	0.098	0.124	0.16
Thigh and Drumstick	18.92	10.96	19.72	12.19	19.18	11.22	19.88	11.01	20.64	10.07	19.66	11.09	6.02	11.19	0.139	0.093	0.299	0.34
Breast Meat	24.22	24.14^bc^	25.99	28.85^a^	25.43	26.95^ab^	26.4	23.59^bc^	26.67	20.90^c^	25.74	24.89	8.44	14.89	0.300	0.100	0.435	1.38

CV=Coefficient of variation, SEM=Standard error of mean, T1=Control, T2=Synbiotics through water, T3=*in ovo* synbiotics, T4=*in ovo* synbiotics+synbiotics through feed, T5=Synbiotics through feed. In the same row, values with the same letter superscripts (a, b, and c,) indicate no significant difference (p > 0.05), while with different superscripts indicate a significant difference (p < 0.05).

### Ileum histomorphometry

**Figure-3:**
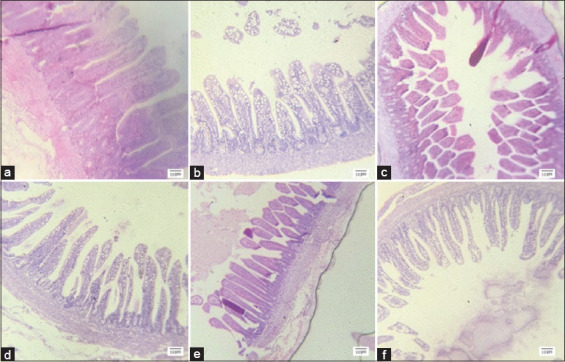
Histomorphology of ileum on day 1 of age. T1-(a and b, control, no use *in ovo* supplements), T2-(c and d, *In ovo* distilled water on 17.5 days) and T3-(e and f, *In ovo* synbiotic on 17.5 days).

**Figure-4:**
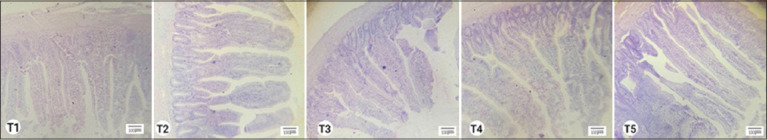
Histomorphology of ileum on 21 days (T1=No use of supplements, T2=synbiotic through water, T3=*In ovo* synbiotic, T4=*In ovo* synbiotic and synbiotic through feed, and T5=Synbiotic through feed only).

**Figure-5:**
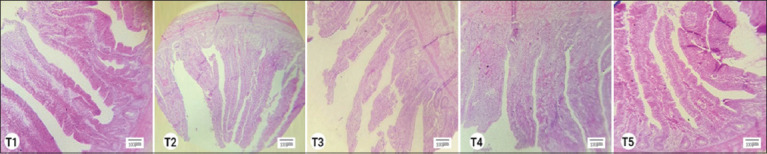
Histomorphology of ileum on 42 days (T1=No use of supplements, T2=synbiotic through water, T3=*In ovo* synbiotic, T4=*In ovo* synbiotic and synbiotic through feed, and T5=Synbiotic through feed only).

Ileum’s histomorphology differed significantly from the control group (431.21 μm). The maximum villus length (652.12 μm) was observed when synbiotics were administered on 21 days. The treatment group, which utilized synbiotics, had a significantly greater VH/CD ratio (4.70) compared to the control group (2.67) after 21 days. In the synbiotic-treated group, the longest villi measured 851.32 μm compared to 743 μm in the control group. The synbiotic treatment given through *in ovo* and regular water supply on 42 days resulted in the longest villi. At the same age, CD and VH/CD ratio were alike. The measurements of villi length, CD, and VH/CD ratio are presented in Table-11. The histomorphologic images of ileum villi of different treatments on days 1, 21, and 42 were taken during the examination of slides are shown in Figures-3, 4, and 5, respectively.

**Table-11:** Ileal histomorphometry of Cobb-500 broilers on days 21 and 42 after administration of synbiotics using various methods.

Treatments	VH, μm		CD, μm		VH/CD	
		
	21 days	42 days	21 days	42 days	21 days	42 days
T1	431.21d	743.76^b^	164.13	179.74	2.67^c^	4.17
T2	546.68^c^	846.75^a^	156.28	185.61	3.55^b^	4.63
T3	603.55^b^	797.43^ab^	137.50	161.90	4.40^a^	4.98
T4	605.82^b^	851.32^a^	147.26	168.27	4.18^a^	5.14
T5	652.12^a^	809.00^ab^	139.29	169.06	4.70^a^	4.85
Average	567.88	809.65	148.89	172.91	3.90	4.75
CV, %	6.10	7.66	12.73	13.52	10.76	13.63
p-value	< 0.001	0.039	0.236	0.425	< 0.001	0.126
SEM (±)	38.04	19.50	5.06	4.27	0.36	0.17

CV=Coefficient of variation, SEM=Standard error of the mean, T1=Control, T2=Synbiotics through water, T3=Synbiotics *in ovo*, T4=Synbiotics *in ovo*+synbiotics through feed, T5=Synbiotics through the feed. In the same row, values with the same letter superscripts indicate no significant difference (p > 0.05), while with different superscripts (a, b, and c) indicate a significant difference (p < 0.05). VH=Villi height, CD=Crypt depth

## Discussion

In livestock production, antibiotics are often given in subtherapeutic doses to enhance growth and safeguard against illness. With antibiotic use becoming more limited, discovering effective alternatives is growing in necessity [30].

Synbiotics, blending probiotics and prebiotics for enhanced animal growth while inhibiting harmful bacteria, offer a productive substitute for in-feed antibiotics [31]. In poultry, synbiotics can be administered through multiple methods. The addition of feed additives, nutrients, hormones, probiotics, prebiotics, or their combinations via *in ovo* techniques has been proven to enhance poultry production. It results in improved growth and feed conversion efficiency, optimal gastrointestinal tract development, increased carcass yield, decreased embryo mortality, and superior immunity [32, 33]. The day-old body weight gain is likely attributed to the injected sterile water and the synbiotic combination. In the final stage, the embryo consumes the amniotic fluid directly. Late hatching of *in ovo* chicks was a result of interfered hatching process. Differences and significance in body weight could be attributed to varying degrees of water loss in eggs that hatch earlier. The intestinal surface area and digestive/absorptive capacities are significantly increased as intestinal brush border membranes, or microvilli, undergo rapid expansion in the final 2 days of incubation [34]. After 17 days, the embryo begins swallowing the amniotic fluid. Commensal bacteria in the intestine ferment prebiotics administered on 17.5 days through *in ovo*. Volatile fatty acids produced during this process, lower the pH of intestinal contents. Butyrate, produced by the microbiota, is one of these fatty acids. Butyrate’s contribution to intestinal epithelial cell growth is evidenced by its positive, significant impacts [35].

*In ovo* feeding with *Lactobacillus acidophilus* and mannan-oligosaccharide, or their combination, significantly enhanced hatch weight without influencing hatchability or livability [36]. Hatchability was negatively influenced by *in ovo* synbiotic [37], which supports the present study findings. Supplying nutrients from outside during embryonic development advances gut development and enhances nutrient digestion and absorption. Ingesting prebiotics and synbiotics through *in ovo* administration modifies intestinal structure, enhancing both VD and CD, thereby augmenting the nutrient-absorbing surface area. These structural modifications boost growth productivity [24]. In the present study, an increase in VH and CD was observed following the *in ovo* injection of synbiotics. Feeding embryos during the incubation period and immediate post-hatch feeding is a critical period for broilers, which can improve gut development, immune system, carcass quality, hatching weight, and epigenetics of the birds [38]. Delay feeding during the early post-hatch period causes underdevelopment of the intestine and reduces muscle growth, resulting in stunted growth of broilers [39]. Immediately after hatching, applying gel droplets containing synbiotic at the hatchery and adding synbiotic to the feed throughout the growth cycle enhances broiler feed efficiency and welfare [40]. A single *in ovo* prebiotic injection given to a chicken embryo can replace the need for post-hatching water supplementation [41]. Prebiotics and synbiotics administered *in ovo* resulted in positive effects on the villi of the duodenum, jejunum, and ileum within 1 day [42]. Day-old chicks gained more weight and had healthier intestines due to *in ovo* feeding of synbiotics.

The incorporation of synbiotics in poultry diets enhances the intestinal beneficial microbes, contributing to the prevention of intestinal issues that support broiler survival. The addition of synbiotics to poultry feed, as reported by Mohammed *et al*. [43], decreases mortality due to heat stress during the summer months. Synbiotics promote intestinal health, enhance immune function, and favorably alter the intestinal microbiota. Synbiotics boost beneficial bacteria populations, such as Lactobacillus and Bifidobacterium, and concurrently minimize harmful bacteria, such as Clostridium and *Escherichia coli*, leading to mortality rate reduction [28, 44–48].

Consuming synbiotics alongside feed can enhance protein, mineral, and vitamin absorption. Synbiotics enhance feed efficiency by altering the microflora composition, growth, and activity within the gastrointestinal tract, improving animal’s growth and health [49]. Feed consumption was not affected during all experimental periods using species of Bifidobacterium *in ovo*, whereas the FCR was increased only for the overall experimental period (1–28 days of age) [50]. Synbiotic levels in feed had a linear effect on feed intake [51]. The control group gained less weight than the synbiotic-treated group [52]. Broiler feeder diets supplemented with any synbiotic concentration resulted in significantly improved body weight gain, feed consumption, and FCR [53], and survival ability compared to control groups [54]. A higher synbiotic concentration and lower pH could stimulate the growth of beneficial bacteria, enhance nutrient absorption and digestion, and ultimately result in increased feed intake and weight gain. In each treatment group, feed consumption was equivalent. The insufficient effect might be due to the use of synbiotic at normal doses in smaller feeding trials, or the non-uniform mixing. The synbiotic-treated group had a higher weight at 6 weeks than the control group. Using synbiotics long-term resulted in the growth of advantageous gut bacteria and benefited overall health.

Synbiotics modify the intestinal microbiota composition with the aid of living beneficial microorganisms [55, 56]. The synbiotic dietary supplement had a more favorable impact on FCR than the control group [57]. The synbiotic and acidifier groups had significantly lower FCRs than the control group. No significant differences in FCRs were found between broiler chickens in the prebiotic/probiotic and control groups [52]. The study’s findings were mirrored during the first 2 weeks with no significant variations, and the FCR was decreased from the 3^rd^ week onwards. Feeding poultry a synbiotic supplement at a rate of 1 g/kg led to remarkable gains in both body weight and feed conversion in comparison to the control group [58]. Supplementing broiler chick diets with probiotics, prebiotics, or synbiotics boosts intestinal morphology, fat metabolism, and immune function, resulting in improved growth performance and overall health benefits [59].

Synbiotics enhanced the blood parameters of broilers. Serum cholesterol levels decreased significantly as a result of the synbiotic dietary supplementation [57]. According to our research, *in ovo* synbiotic and synbiotic through feed groups had lower cholesterol levels (106.50 mg/dL) than the control (119.33 mg/dL). The control group’s blood cholesterol level was higher than that of the synbiotic group [60]. Groups given probiotics had significantly lower serum cholesterol, triglycerides, and overall, lipid levels than the control group. *Lactobacillus plantarum* and *Lactobacillus lactis* supplementation improves broiler physiology [61]. The inclusion of mannano-ligosaccharides in the broiler’s diet decreases cholesterol levels with no impact on feed intake or live weight gain [62].

Probiotics aid in reducing serum cholesterol by enzymatically deconjugating bile acids, converting cholesterol to non-absorbable coprostanol and synthesizing new, and cholesterol-eliminating bile acids. Prebiotics enhance intestinal thickness and viscosity, promoting decreased cholesterol absorption and increased hepatic cholesterol catabolism. Prebiotics boost liver’s short-chain fatty acid production, thus hindering cholesterol and triglyceride synthesis [63, 64]. Synbiotic intakes in broilers lead to reduced lipid and cholesterol levels in their bloodstream [65, 66]. 1.5 g/kg of synbiotic significantly raised blood glucose and lowered cholesterol levels [58]. In addition, compared with the control, decreased serum total cholesterol levels and low-density lipoprotein cholesterol were found after 28 and 42 days [59] which all supports the present findings.

In this study, broilers fed synbiotics showed an enhanced dressing percentage. The dressing percentage of the synbiotic group was statistically greater than that of both the control and probiotic-only groups [67]. Discrepant results emerged when broilers received synbiotic in varying quantities (700, 1200, 1700, or 2200 g/t of feed) without notable alterations in carcass features [68]. Dressing percentage, carcass percentage, heart weight, liver weight, gizzard weight, wing percentage, breast percentage, back percentage, thigh percentage, and drumstick percentage in broiler birds have remained largely unchanged [69]. The impact of different prebiotic delivery routes (*in ovo*, in water, and *in ovo* + in water) on slaughter performance and meat quality traits in broiler chickens [70] were as similar with the current results, where there was a greater final body weight in the treatment group than the control, irrespective of the delivery method. *In ovo* or a combination of *in ovo* and in-water prebiotics led to greater carcass weights than those in the in-water group. *In ovo* prebiotic administration resulted in a higher carcass yield than in-water application. Prebiotics and probiotics can enhance body weight gain by boosting feed conversion and improving physiological well-being through mechanisms such as competitive exclusion, enhanced mineral absorption, secretion of digestive enzymes, improved nutrient digestion, and modulated immune status [71].

There are contradictory findings on the effect of synbiotics on the accumulation of belly fat in broilers. For example, some studies suggest that synbiotics do not significantly impact poultry belly fat [29, 72]. Whilst, one of the studies indicated synbiotics can boost broiler performance, economic gain, and feed conversion by reducing abdominal fat [37]. This research clearly indicates a potential link between synbiotic supplementation and a decrease in visible fat in broiler carcasses.

On day 21, the percentage of visible fat remained unchanged in this study. The difference in visible fat among the treatments could be due to the diet’s feed composition, which contains less oil in the initial phases than in the final phases. On day 42, the control group had a higher average (3%) versus the treatment groups (approximately 2.56%, with the lowest values in groups 3 and 4 using synbiotic). Abdominal fat percentage was lower in the synbiotic-supplemented group [57]. Using synbiotics led to a significant reduction of abdominal fat in broilers [73]. Both ages yielded more drumstick and breast meat than the control group at all stages, except on 42 days. In the synbiotic-treated group, the small intestine weighed less than in the control group during the current experiment. On the contrary, where the weight of the small intestine remained greater for probiotic (3.17%) or synbiotic-fed birds (3.11%) than for controls (2.89%), but the relative weight of the liver was found lower (1.87%) in the synbiotic-treated group than in the control group (2.04%) (p < 0.1) [67].

Synbiotic administered *in ovo* led to better growth, enhanced immunity, improved small intestine morphology, and beneficial effects on cecal microflora [24]. The *in ovo* group treated with bifidobacteria had an enhanced ileal architecture, as evidenced by the highest values of CD/villus height and villus height recorded in *Bacillus* *animalis* (936.6 μm and 11.80), surpassing those of the control group (537.1 μm and 6.93) [42]. Histological examination revealed a significant increase (p < 0.001) in the ratio of the height of the villus to the depth of the crypt (7.13 0.1) and the actual height to CD (7.13 ± 0.1) and actual villus height (774 ± 9 μm) in the ileum when synbiotic was introduced, compared with the control group (614 ± 9 μm, 4.86±0.1). On the contrary, the depth of the ileal crypts decreased with the supplementation with synbiotic (117 ± 2 μm) compared to the control group (128 ± 2 μm) [74], which agrees with the present findings.

0.1% synbiotic supplementation in feed led to a decrease in ileal villus height and an increase in ileal villus width and surface area [11]. On day 21, birds fed *Paenibacillus xylanexedens ysm1* and lactulose-supplemented diets showed significant growth in ileum villus height and CD. In birds fed probiotic and synbiotic diets, the ileum villus height was significantly greater on day 21 compared to the control. On day 42, the ratio of VD to CD in birds fed synbiotic diets [48] was uniformly greater, corroborating this research. The broilers given 2.5 g/kg of synbiotic through in feed administration had significantly longer villi than the other groups [75]. 28-day-old broilers supplemented with synbiotics or prebiotics had a greater jejunal villus height [59]. On 42 days, the ileum villus height significantly escalated due to synbiotic supplementation in the broiler diet [76]. Several studies [67, 77–80] reported a noteworthy enhancement in villus height by incorporating dietary probiotics and prebiotics, leading to a notable rise in villus height in the duodenum and ileum, as well as in the CD ratio [81]. In the ileum, the control group showed greater CD than the synbiotic group [67]. The length and depth of the ileal villi were comparatively higher in the early days on day one [42], day 14, and day 21 [24], which is consistent with the findings of the present study, where the depth of the ileal crypt and the height of the villi were more prominent in the early days of age. Probiotics increased villus height and decreased CD [52, 82, 83], in association with prebiotics as our results confirm.

## Conclusion

The study revealed that utilizing synbiotics, regardless of administration method, enhances broiler performance. The use of synbiotic has resulted in decrease in FCR, intestine and liver weight, improvements in dressing percentage, and favorable changes in gut histomorphometry in the ileum. Detailed investigations are required into gut flora, bacterial challenges, intestinal pH shifts, and synbiotic’s economic feasibility. Under the right management conditions, the *in ovo* method combined with other synbiotic feeding methods yielded better results for hatchability and broiler performance, indicating that synbiotics may serve as an alternative to preventive antibiotic use for gut health management.

## Authors’ Contributions

AA: Collected data, performed statistical analysis, and prepared the manuscript. BD: Supervised the project and revised the manuscript. HBB: Supervised the project and supported field and lab work. SRB: Developed the protocol, supported data analysis, and manuscript revision. All authors have read, reviewed, and approved the final manuscript.
